# Toward a Treatment Goal Framework for Emerging Therapies in IgAN: Implications for Future Research

**DOI:** 10.1016/j.ekir.2026.107000

**Published:** 2026-08-01

**Authors:** Muh Geot Wong, Jiahua Li, Chi Chu, Manish Maski, Richard Lafayette

**Affiliations:** 1 Department of Renal Medicine, Concord Repatriation General Hospital, Concord, New South Wales, Australia; 2 Concord Clinical School, University of Sydney, Concord, New South Wales, Australia; 3 Vertex Pharmaceuticals Incorporated, Boston, Massachusetts, USA; 4 Division of Nephrology, Department of Medicine, Stanford University, Stanford, California, USA

The therapeutic landscape for IgA nephropathy (IgAN) is evolving rapidly, with new agents and mechanistic insights reshaping how clinicians and investigators think about disease modification, biomarker interpretation, and treatment strategies. The recent Kidney Disease: Improving Global Outcomes IgAN interim commentary^1^ builds a structured framework for organizing therapies according to the treatment goals they address. These goals include stopping the synthesis of pathogenic forms of IgA and/or IgA-containing immune complexes (Hits 1–3 of IgAN pathogenesis), stopping IgA and/or IgA-containing immune complex-mediated kidney injury (Hit 4), and managing the generic responses to IgAN-induced nephron loss. This framework helps clarify the mechanistic and clinical implications of emerging therapies in the broader context of IgAN care, while also highlighting a broader challenge—the field is changing faster than traditional guideline cycles can accommodate. Several observations about mechanism, proteinuria, and hematuria therefore support consideration of a more agile, trigger-based approach to future guideline updates.

The first observation is that many therapies exert clinical effects which span multiple pathogenic hits of IgAN. This broad effect may arise from nonspecificity, as seen with systemic glucocorticoids, which affect multiple inflammatory pathways but are associated with significant toxicity limitations. Alternatively, they may reflect the consequences of intervening at an upstream pathogenic step, even when the therapy does not directly target downstream pathogenic processes, such as reducing glomerular inflammation. This “convergence” of hits requires further study to understand the interplay between upstream disease drivers and downstream clinical manifestations. Viewed through the treatment goal framework, this distinction is especially important because therapies may differ not only in where they act within IgAN pathogenesis, but also in where their clinical effects are ultimately observed.

Emerging therapies illustrate this mechanistic diversity. B cell-modulating therapies act on upstream immunologic drivers involved in the generation of pathogenic IgA and IgA-containing immune complexes, whereas complement-directed therapies such as iptacopan target downstream pathways of immune complex-mediated kidney injury.^2^^,^^3^ Other therapies, such as renin-angiotensin system blockers, sodium-glucose cotransporter 2 inhibitors, and endothelin receptor antagonists, are directed less at the core immunopathogenesis itself and more at the generic responses to IgAN-induced nephron loss.^4^ These distinctions are important because clinical manifestations such as proteinuria, hematuria, and estimated glomerular filtration rate decline may improve in response to therapies mapped to different treatment goals within the framework. Mechanistic heterogeneity also exists within therapeutic classes that adds further complexity. For instance, the B cell targeting therapies include a proliferation-inducing ligand-only inhibition (e.g., sibeprenlimab) and dual B cell-activating factor and/or a proliferation-inducing ligand inhibition (e.g., povetacicept), among other approaches that target humoral immunity.^2^^,^^5^ Whether these mechanistic distinctions translate into meaningfully different clinical response profiles—and how they should inform treatment sequencing and combination strategies for optimal outcomes in IgAN—are questions that ongoing Phase 3 programs are well positioned to address, with direct implications for the pace and scope of future guideline revision.

The second observation concerns proteinuria as a marker of treatment efficacy. The percentage reduction in proteinuria over approximately 9 months has been established as a reasonably likely surrogate end point for progression to kidney failure in IgAN^6^^,^^7^ and has underpinned recent accelerated approvals, with confirmatory kidney function data (e.g., estimated glomerular filtration rate slope) needed to demonstrate clinical benefit for full approval. As the field advances, emerging research questions concern not only the comparative magnitude of proteinuria reduction, but also its kinetics and quality—How rapidly does proteinuria decline? How deep is the reduction? Specifically, to what extent do therapies achieve the Kidney Disease: Improving Global Outcomes treatment targets of proteinuria < 0.5 g/d (ideally < 0.3 g/d)? How durable is this response over time? Different therapeutic mechanisms may demonstrate distinct response profiles across these dimensions. Understanding the speed, depth, and durability of proteinuria reduction and how these characteristics relate to meaningful long-term kidney outcomes across and within therapeutic classes represents an important priority for ongoing Phase 3 programs. These features of proteinuria response may ultimately refine how optimal treatment response is interpreted in IgAN and help distinguish between therapies that achieve similar average reductions in proteinuria but differ in the timing or sustainability of benefit. As the evidence base matures and clinically meaningful response features are better characterized, future guidelines may need to incorporate these nuances into recommendations regarding monitoring, prognosis, and treatment modification.

The third observation concerns hematuria, an indicator of active inflammation and glomerular injury. Historically, its value as a treatment biomarker in IgAN has been incompletely defined, and it has not featured prominently in guideline discussions to the same degree as proteinuria. The TESTING and STOP-IgAN trials both showed reductions in hematuria, but yielded different clinical outcomes.^8^, ^9^, ^10^ Rather than arguing against the relevance of hematuria itself, these findings may underscore the challenges of interpreting this biomarker across studies that differed substantially in design, patient populations, treatment regimens, and follow-up duration. For example, the immunosuppression arm of STOP-IgAN adopted a treatment strategy that varied according to kidney function and was conducted in a White European population, whereas the TESTING trial evaluated oral methylprednisolone in a predominantly Asian population. More recent data from global, pivotal, randomized controlled studies may provide a clearer and more interpretable picture of hematuria as a marker of treatment response, by virtue of more standardized study designs, longitudinal hematuria assessment, and placebo-controlled comparisons. This may be especially relevant when the therapeutic mechanisms under study are more specifically targeted to IgAN pathogenesis, as this could help clarify whether changes in hematuria track with modulation of active disease. The clinical question is therefore becoming more nuanced—not simply whether hematuria has clinical utility, but how it should be interpreted in relation to active disease, therapeutic mechanism, and treatment goals. Standardized hematuria measurement and its consistent inclusion as a secondary end point across ongoing Phase 3 programs will further refine our understanding of its utility as a biomarker for IgAN treatment. As these relationships become clearer, future guideline updates may be able to more explicitly define how hematuria fits within the treatment goal framework as a marker of active glomerular inflammation and a potential treatment-responsive feature.

The Kidney Disease: Improving Global Outcomes interim commentary^1^ is a timely acknowledgment that the IgAN therapeutic landscape is changing faster than traditional guideline cycles can accommodate. Multiple accelerated approvals have occurred since the publication of the most recent 2025 guideline, and additional Phase 3 readouts are anticipated within the next 18 to 24 months. Consequently, updates may be required not only for newly approved therapies, but also for emerging evidence that refines interpretation of treatment goals, biomarkers, and therapeutic sequencing. In practice, triggers would need to be prespecified evidence milestones with clear potential to change recommendations, such as pivotal readouts, regulatory approvals, and biomarker validation milestones. A trigger-based approach could complement existing Kidney Disease: Improving Global Outcomes guideline methodology by enabling focused updates when predefined evidence thresholds are reached. Under this model, guidance could evolve through targeted, evidence-driven revisions while preserving the rigor and transparency that underpin guideline development. Rather than waiting for accumulated evidence to justify a full guideline revision, focused updates could be initiated when new information has clear potential to influence clinical practice. This “living” guideline approach would mean maintaining guidance continuously through focused, evidence-driven updates as new data emerge, while preserving the rigorous evaluation needed to ensure recommendations remain high quality and well supported by evidence. In turn, this will ensure that new evidence translates rapidly and consistently into clinical practice and, ultimately, into meaningful benefit to patients with IgAN.
